# Preemptive Infiltration with Betamethasone and Ropivacaine for Postoperative Pain in Laminoplasty or Laminectomy (PRE-EASE): study protocol for a randomized controlled trial

**DOI:** 10.1186/s13063-020-04308-z

**Published:** 2020-05-05

**Authors:** Niti Shrestha, Liang Wu, Xiaodi Wang, Wenqing Jia, Fang Luo

**Affiliations:** 1 Department of Pain Management, Beijing Tiantan Hospital, Capital Medical University, Beijing, 100050 China; 2 Department of Neurosurgery, Beijing Tiantan Hospital, Capital Medical University, Beijing, China

**Keywords:** Betamethasone, Diprospan, Preemptive infiltration, Postoperative pain, Laminoplasty, Laminectomy, Protocol, Randomized controlled trial

## Abstract

**Background:**

Laminoplasty and laminectomy have been used for decades for the treatment of intraspinal space-occupying lesions, spinal stenosis, disc herniation, injuries, etc. After these procedures, patients often experience severe postoperative pain at the surgical site. Intense immediate postoperative pain after many spinal procedures makes its control of utmost importance. Preemptive injection of local anesthetics can significantly reduce postoperative pain during rest and movement; however, the analgesic effect is only maintained for a relatively short period of time. Whether betamethasone combined with local anesthetic for laminoplasty or laminectomy has better short-term and long-term effects than the local anesthetic alone has not been reported yet.

**Methods:**

The PRE-EASE trial is a prospective, randomized, open-label, blinded endpoint, single-center clinical study including 116 participants scheduled for elective laminoplasty or laminectomy, with a 6 months’ follow-up process. Preemptive local infiltration with betamethasone and ropivacaine (treatment group) or ropivacaine alone (control group) throughout the entire thickness of the planned incision site will be performed by the surgeon prior to making the incision. The primary outcome will be the cumulative butorphanol consumption within the first 48-h postoperative period.

**Discussion:**

This study will add significant new knowledge to the effect and feasibility of preemptive local infiltration of betamethasone for postoperative pain management in laminoplasty and laminectomy.

**Trial registration:**

ClinicalTrials.gov: NCT04153396. Registered on 6 November 2019.

## Administrative information

Note: the numbers in curly brackets in this protocol refer to Standard Protocol Items: Recommendations for Interventional Trials (SPIRIT) checklist item numbers. The order of the items has been modified to group similar items (see http://www.equator-network.org/reporting-guidelines/spirit-2013-statement-defining-standard-protocol-items-for-clinical-trials/).

**Table:** 

Title {1}	*Pre*emptive Infiltration with B*e*tamethasone *a*nd Ropivacaine for Po*s*top*e*rative Pain in Laminoplasty or Laminectomy (PRE-EASE): A Study Protocol for a Randomized Controlled Trial
Trial registration {2a and 2b}	ClinicalTrials.gov, NCT04153396. Registered on 6 November 2019 *https://www.clinicaltrials.gov/ct2/show/NCT04153396*
Protocol version {3}	2020/03/10 Protocol Version 3.0
Funding {4}	Beijing Municipal Administration of Hospitals Clinical Medicine Development of Special Funding Support (grant no. ZYLX201708).
Author details {5a}	Niti Shrestha*, Department of Pain Management, Beijing Tiantan Hospital, Capital Medical University, Beijing, China. Liang Wu*, Department of Neurosurgery, Beijing Tiantan Hospital, Capital Medical University, Beijing, China. Xiaodi Wang*, Department of Pain Management, Beijing Tiantan Hospital, Capital Medical University, Beijing, China. Wenqing Jia, Department of Neurosurgery, Beijing Tiantan Hospital, Capital Medical University, Beijing, China. Fang Luo, Department of Pain Management, Beijing Tiantan Hospital, Capital Medical University, Beijing, China.
Name and contact information for the trial sponsor {5b}	Beijing Municipal Administration of Hospitals Clinical Medicine Development of Special Funding Support Contact information: 008613661058642
Role of sponsor {5c}	The funders have no role in the design, data collection and analysis, decision to publish or the preparation of the manuscript

*Signify 3 co-first authors who contributed equally to this work

## Introduction

### Background and rationale {6a}

Laminoplasty and laminectomy have been used for decades for the treatment of intraspinal space-occupying lesions, spinal stenosis, disc herniation, injuries, etc. After these procedures, patients often experience severe postoperative pain at the surgical site. With currently available systemic analgesics, the drug-related side effects may exacerbate when the drug concentration in the blood is high. However, when the blood concentration is low, there may be insufficient analgesia which may also lead to insufficient management of pain at movement [1]. Intense immediate postoperative pain after many spinal procedures makes its control of utmost importance [2]. Despite recent advancements in postoperative pain management, there is evidence of inadequate postoperative pain control after spinal surgery, which leads to reduced patient mobility [1, 2]. Early mobilization after spine surgery is vital for reduction of hospital stay and postoperative complications, and better performance-based functional tests and patient-reported outcome measures [3]

Severe immediate postoperative pain increases the risk of chronic pain along with the occurrence of nerve injury and the development of neuronal plasticity associated with peripheral and central sensitization [4]. Central sensitization, an increase in central nervous system excitability, occurs due to the ongoing noxious input [5], which leads to allodynia, the perception of pain resulting from a normally non-painful stimulus [6]. Therefore, reducing postoperative acute pain is vital for the prevention of chronic pain.

At present, several pain-controlling methods are available, with opioids being the cornerstone for management of severe acute postoperative pain [2, 7]. However, there are many compelling reasons to avoid opioids in surgical patients due to their numerous side effects [8]. Methods for systemic administration include oral analgesics, intermittent intravenous or intramuscular injections, and patient-controlled intravenous analgesia, etc. [1]. Nevertheless, the aforementioned methods may have many side effects, and are usually used after the occurrence of pain. Hence, the analgesic effects are sometimes inadequate.

Topical administration options have fewer systemic side effects. Preemptive injection of local anesthetics can significantly reduce postoperative pain, although the analgesic effect is maintained for a relatively short period of time. Incidences of technical failure or local anesthetic toxicity from wound catheters were found to be low in a study by Liu et al. [9], although other reports have raised concerns about probable wound infection from the existence of a catheter [10]. Furthermore, indwelling catheters come with a risk of complications such as prolapse or obstruction of the catheter. Cost-effectiveness, optimal site for catheter placement, and optimal dosage are also factors to be considered [9]. Techniques such as epidural analgesia and nerve blockade may have a possible high failure rate and not be cost effective, but they can deliver better postoperative analgesia [11–13]. Gurbet et al. [1] reported that preemptive infiltration of bupivacaine or levobupivacaine combined with methylprednisolone, a short-acting glucocorticoid, can effectively control pain after unilateral lumbar laminectomy. However, this solution has a shorter duration of action, and the study involved merely 24-h postoperative observation and only 60 participants. Ersayli et al. [14] reported that, compared to infiltration at wound closure, preemptive injection of bupivacaine or bupivacaine-methylprednisolone into muscles near the incisional site provided more effective analgesia after lumbar discectomy, and they concluded that methylprednisolone combined with local anesthetic was not superior to the analgesic effect of local anesthetic alone. However, in the study [14], 75 participants were enrolled with only 15 in each group. Therefore, it is necessary to observe more cases to explore other compatibilities of drugs with longer duration of action and stronger analgesic effect.

Betamethasone, a stereoisomer of dexamethasone, is a long-acting corticosteroid which has longer lasting anti-inflammatory properties because of its partial presence in particulate form in ropivacaine, acting as a local reserve [15]. Whether betamethasone combined with local anesthetic for laminoplasty or laminectomy has better analgesic effects than the local anesthetic alone has not been reported yet. Therefore, a detailed study is needed to compare the postoperative analgesic efficacy of preemptive infiltration of betamethasone plus ropivacaine and ropivacaine alone for laminoplasty or laminectomy.

### Objectives {7}

We hypothesize that preemptive local infiltration of betamethasone plus ropivacaine helps relieve postoperative pain, reduces the request for postoperative analgesics, and promotes early rehabilitation without significant risks.

### Trial design {8}

**Fig. 1:**
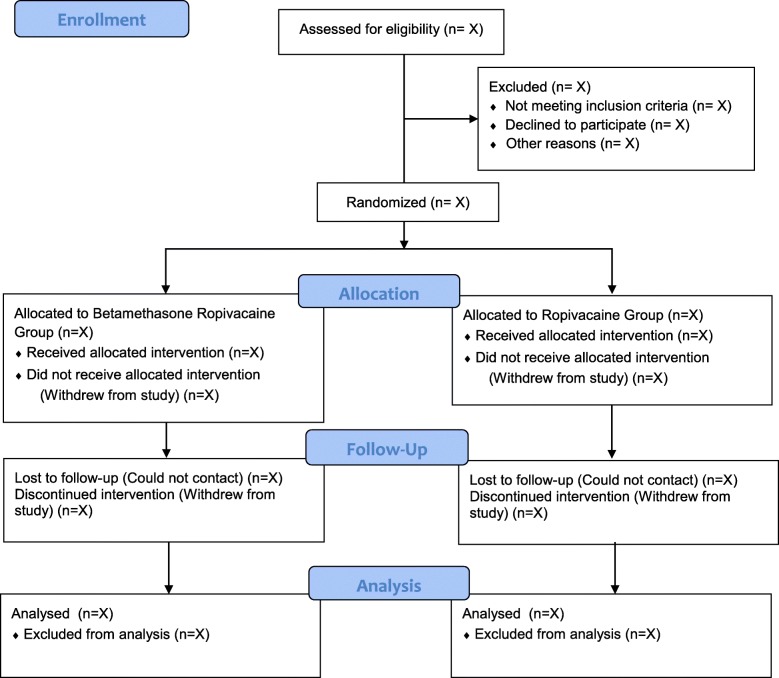
CONSORT patient flow diagram of the PRE-EASE trial

The PRE-EASE trial is a prospective, randomized, open-label, blinded endpoint (PROBE), single-center clinical study designed to compare the postoperative analgesic efficacy of preemptive wound infiltration of ropivacaine alone and betamethasone plus ropivacaine for laminoplasty or laminectomy. In total, 116 patients will be randomly assigned to the betamethasone-ropivacaine (treatment) group and the ropivacaine (control) group at a 1:1 ratio. The Consolidated Standards of Reporting Trials (CONSORT) patient flow diagram is presented in Fig. 1.

## Methods: participants, interventions, and outcomes

### Study setting {9}

This is a single-center study which will be conducted from January 2021 to June 2022 at Beijing Tiantan Hospital, Capital Medical University, Beijing, China.

### Eligibility criteria {10}

#### Inclusion criteria

The inclusion criteria are as follows:

- Patients scheduled for laminoplasty or laminectomy
- American Society of Anesthesiologists (ASA) classification of I or II
- Age 18 to 64 years
- Participants with an anticipated full recovery within 2 h postoperatively.

#### Exclusion criteria

Potential participants will have to be excluded if they:

- Refuse to participate
- Cannot use a patient-controlled analgesia (PCA) device and cannot understand the instructions of a Visual Analog Score (VAS)
- Have previous history of spinal surgery
- Are allergic to opioids, betamethasone, or ropivacaine
- Have peri-incisional infection
- Have history of stroke or a major neurological deficit
- Have trauma, deformity
- Have psychological problems
- Have extreme body mass index (BMI) (< 15 or > 35 kg/m^2^)
- Have history of excessive alcohol or drug abuse, chronic opioid use (more than 2 weeks), or use of drugs with confirmed or suspected sedative or analgesic effects
- Are using systemic steroids
- Are pregnant or breastfeeding
- Have preoperative Glasgow Coma Scale score < 15
- Have received radiation therapy or chemotherapy preoperatively, or with a high probability to require postoperative radiation therapy or chemotherapy according to the preoperative imaging
- Are not able to give written informed consent.

### Who will take informed consent? {26a}

Participants will be recruited from the neurosurgical outpatient department at Beijing Tiantan Hospital, affiliated to Capital Medical University, Beijing, China, by two research members from the department of neurosurgery. Based on the inclusion and exclusion criteria, patients will be screened for study participation. Patients scheduled for surgery who fulfill the eligibility criteria and express an interest in participating in the study will be visited by a research assistant, 1 day before the surgery, to obtain written consent. A verbal explanation of the written consent will be provided by the research member, and any questions regarding the study will be answered. Each participant will have sufficient time to decide whether to participate in this study. If patients are willing to participate, written consent will be obtained.

### Additional consent provisions for collection and use of participant data and biological specimens {26b}

On the consent form, participants will be asked if they agree to the use of their data, should they choose to withdraw from the trial. Participants will also be asked for permission for the research team to share relevant data with people from regulatory authorities, where relevant. This trial does not involve collecting biological specimens for storage.

### Interventions

#### Explanation for the choice of comparators {6b}

A total of 30 ml of solution will be prepared for each group, which will include 0.5 ml of compound betamethasone injection (Diprospan® betamethasone propionate 5 mg and betamethasone sodium phosphate 2 mg) added to 14.5 ml of saline and 15 ml of 1% ropivacaine (NaiLePin®10 mg/ml, AstraZeneca AB, Södertälje, Sweden) for the treatment group and 15 ml of ropivacaine added to 15 ml of saline for the control group [15, 16]. The study investigator will be responsible for preparing the respective drugs to be used for preemptive infiltration in these two groups, and the neurosurgeon will infiltrate the planned incision site with the respective study solution, prior to the incision.

### Intervention description {11a}

#### Preemptive infiltration

A 10-cm-long 22-gauge needle will be introduced into the planned incision site by the surgeon, to infiltrate the prepared solution. A total of 10 ml of solution will be injected into each level. The total volume of solution to be injected will be based on the number of levels to be treated, will be consistent in every participant, and will be recorded by the investigator. The study solution will be injected into the subcutaneous tissue and paravertebral muscles, along with the posterior area around the spinous process, lamina, transverse process, and the facet joints, along both sides of the planned incision. The epidural space and intrathecal space will not be infiltrated. The local infiltration solution in the treatment group will consist of betamethasone and ropivacaine with saline, whereas ropivacaine alone will be used with saline in the control group. All other aspects of the rehabilitation process will be identical between the two groups.

#### Anesthesia management

During the preoperative visit, after signing the written consent, patients will be taught how to indicate postoperative pain based on a VAS, with levels ranging from 0 (no pain) to 10 (maximal pain). Patients will also be taught how to use the PCA device. In the operating theater, each patient will be prepared for monitoring of continuous blood pressure and heart rate, peripheral pulse oximetry, bispectral index (BIS system, Covidien/Medtronic, Minneapolis, MN, USA), and electrocardiography. Then a peripheral venous cannula will be inserted and an intravenous (IV) infusion of crystalloid solution will be started. Each participant will be premedicated with an IV infusion of midazolam 0.03 mg/kg before the induction of general anesthesia. A standard general anesthesia protocol will be followed, using 0.3–0.4 μg/kg sufentanil, 1.5–2 mg/kg propofol, and 0.2 mg/kg cisatracurium or 0.6 mg/kg rocuronium. Anesthesia will be maintained with IV administration of propofol and remifentanil, and muscle relaxation will be maintained using IV cisatracurium or rocuronium. After endotracheal intubation, invasive blood pressure will be monitored by placing an arterial line if deemed necessary by the anesthesiologist in charge. Ventilation will be adjusted as needed to maintain normocapnia. Anesthesia will be maintained with IV propofol and remifentanil, and muscle relaxation will be maintained using IV cisatracurium or rocuronium. Local infiltration of the prepared solution will be performed by the neurosurgeon, before the incision is made. Sufentanil will be administered at certain time points to attenuate potent stress responses and maintain the mean arterial pressure and heart rate fluctuations within a 20% range of baseline. No additional analgesics will be administered intraoperatively. Antihypertensive drugs or vasoactive drugs will be administered as needed, and crystalloid and colloid solutions will be infused as necessary, by the anesthesiologist in charge. All intraoperative physical parameters, fluid input and output, and dosage of all drugs will be closely monitored and recorded.

#### Additional interventions

The ongoing continuous infusion of propofol and remifentanil will be stopped right after the suture of the incision. To prevent postoperative nausea and vomiting, 8 mg of ondansetron will be administered. Any residual muscle relaxation will be antagonized by atropine and neostigmine. Once the patient is hemodynamically stable, along with the recovery of adequate spontaneous ventilation and satisfactory neurological evaluations, the patient will be extubated and transferred to a post-anesthesia care unit. A PCA device containing 10 mg butorphanol tartrate injection (HengRui Medical Co., Ltd., Lianyungang, Jiangsu, China) and 10 mg tropisetron hydrochloride injection (Southwest Pharmaceutical Co., Ltd., Chongqing, China) diluted to a total volume of 100 ml with 0.9% normal saline, will be connected to the patients, for a total duration of 48 h. Apon® electronic infusion pumps (ZZB-I-150, Apon Medical Technology Co., Ltd., Jiangsu, China) will be used for patient-controlled analgesia (PCA). The PCA device will have a bolus dose of butorphanol set at 0.3 mg with a lockout interval of 15 min. Both the initial dose and background infusion of the PCA device will be set at zero. The participants will be advised to push the analgesic demand button when they feel pain. Each press will be recorded by an electronic memory system, including both valid and invalid presses. Invalid presses refer to presses for bolus during the lockout period. In case of inadequate analgesia four times after the butorphanol bolus, the bolus dose would be increased gradually with the final maximum dose not exceeding 4 mg per hour. There will be real-time updates of drug dosage, press counts, and time of each press to the iPainfree online recording system. Participants will be allowed to take oral supplementary acetaminophen 500–1000 mg every 4 to 6 h if necessary after 48 h, until the end of our study.

### Criteria for discontinuing or modifying allocated interventions {11b}

A detailed recording of all adverse events (AEs) throughout the course of the study will be properly recorded, closely monitored, and reported to the ethics committee as soon as possible, with the intentions of a resolution or stabilization, or even termination of the study if necessary.

### Strategies to improve adherence to interventions {11c}

During the study, periodic monitoring will be conducted by a blinded research member, who will visit each patient in person at 2, 4, 8, 24, and 48 postoperative h, for the recording of postoperative parameters and to ensure adherence to interventions. The blinded postoperative care nurses of the neurosurgical department will be checking on patients from time to time and will advise them to press the analgesic demand button on the PCA device if necessary. Preoperatively, the patients will have been instructed on how to use the PCA device after their written consent is obtained. Both groups will have the PCA device bolus dose of butorphanol set at 0.3 mg with a lockout interval of 15 min, with the background infusion set to zero. There will be real-time updates of drug dosage, press counts, and time of each press, which will also help with monitoring and improving adherence. After the initial 48-h postoperative period and until the end of the study, patients will be allowed to take oral supplementary acetaminophen 500–1000 mg every 6 h if necessary. Follow-up will be conducted on day 3, at weeks 1, 2, 4, and 6, and at months 3 and 6 by a blinded research member.

### Relevant concomitant care permitted or prohibited during the trial {11d}

In the immediate 48-h postoperative period, all participants will be provided with a butorphanol tartrate and tropisetron hydrochloride intravenous PCA device. After 48 h the participants will be allowed to take oral supplementary acetaminophen 500–1000 mg every 4 to 6 h if necessary, until the end of the 6 months’ follow-up period.

### Provisions for post-trial care {30}

There is no anticipated harm and compensation for trial participation.

### Outcomes {12}

Clinical and demographic characteristics such as gender, age, weight, BMI, ASA status, type of surgery (laminoplasty or laminectomy), level of spine to be treated (cervical, thoracic, lumbar, or sacral), number of levels to be treated (1, 2, 3, 4 levels or more than 4 levels), and Oswestry Disability Index (ODI) will be recorded. After the operation, the duration of surgery, length of incision (in millimeters), and volume of local anesthesia (in milliliters) injected for preeemptive infiltration will be recorded. Postoperative complications such as postoperative pain due to spinal cord or nerve injury, wound infection, wound hematoma, delirium, serious adverse effects, and death may affect the follow-up process. Other complications such as allergic reaction, local or systemic toxicity, changes in wound healing, or increased wound drainage will be closely monitored.

### Postoperative recording parameters for up to 48 h

The parameters will be recorded at 2, 4, 8, 24, and 48 h after surgery by a research member, who will visit each patient in person. Pain scores will be measured using the VAS score: an 11-point VAS score during movement (VAS_M_) and at rest (VAS_R_) will be recorded, with 0 indicating no pain and 10 indicating the most severe pain imaginable.

Time of first analgesic demand will be indicated by the first press of the analgesic demand button on the PCA device. The time of first analgesic demand, total press count, the cumulative butorphanol dose for four separate periods (0–4, 4–8, 8–24, and 24–48 h), and total butorphanol dose at 48 h will be recorded.

The Patient Satisfaction Score (PSS) used in this study will comprise points 1–4, based on the study by Mobbs et al. [17].

Postoperative nausea and vomiting (PONV) and Ramsay Sedation Scale (RSS) scores will also be recorded. PONV will be measured using an ordinal scale with the following values: 0 no nausea, 1 mild nausea not requiring treatment, 2 nausea requiring treatment, 3 vomiting. The RSS uses a 6-point scale to assess sedation levels, with 1 indicating agitated, anxious; 2 cooperative; 3 only responds to commands; 4 strong response to glabellar tapping or noisy stimulants; 5 weak response to glabellar tapping or noisy stimulants; 6 no response.

### Postoperative follow-up data recording

Follow-up will be conducted on day 3, at weeks 1, 2, 4, and 6, and at months 3 and 6 by an experienced research member blinded to the study. All the participants will complete a 6 months’ follow-up. The postoperative follow-up data recording parameters will also include VAS and PSS.

World Health Organization Quality of Life-BREF (WHOQOL-BREF) scores will be used to obtain scores for four domains related to quality of life: physical health (7 items), psychological (6 items), social relationships (3 items), and environment (8 items). This assessment will also include two stand-alone questions on overall quality of life and satisfaction with health. Each question will be rated on a scale of 1–5, with higher scores signifying better quality of life.

Functional disability will be assessed preoperatively and at 4 and 6 weeks and 3 and 6 months after surgery using the ODI. The ODI includes 10 questions about pain and activities of daily living. Each item has five response categories, ranging from no pain-related disability (0), to the worst possible pain-related disability (100).

The Patient and Observer Scar Assessment Scale (POSAS), comprised of subjective symptoms of pain and pruritus, will be assessed at 6 months postoperatively.

AEs such as nausea, vomiting, and steroid-related adverse effects (gastrointestinal bleeding, gastritis, delayed wound healing, etc.) will be documented for comparison of outcome.

### Primary outcome

The primary outcome will be the cumulative butorphanol dose during the 48 h after surgery via the PCA device.

### Secondary outcomes

The secondary outcomes are as follows:

1. VAS_M_ and VAS_R_ 2 h, 4 h, 8 h, 24 h, 48 h, 72 h, 1 week, 2 weeks, 4 weeks, 6 weeks, 3 months, and 6 months after surgery
2. Cumulative butorphanol dose for four separate periods (0–4, 4–8, 8–24, and 24–48 h), a total press count including both valid and invalid presses, first analgesia demand on the PCA device
3. PSS 2 h, 4 h, 8 h, 24 h, 48 h, 72 h, 1 week, 2 weeks, 4 weeks, 6 weeks, 3 months, and 6 months postoperatively
4. PONV and RSS scores 2 h, 4 h, 8 h, 24 h, and 48 h after surgery
5. WHOQOL-BREF scores preoperatively and 6 months postoperatively
6. Functional disability assessed by ODI scores preoperatively and at 4 weeks, 6 weeks, 3 months, and 6 months after surgery
7. Wound healing situation assessed by the POSAS scores at 6 months postoperatively.

### Participant timeline {13}

The enrollment, interventions, assessments, and study visits of the PRE-EASE trial are presented in Table 1.

**Table 1:** Study visits of the PRE-EASE trial

	Study period															
	Enrollment	Allocation	Post-allocation													
Time points	Preoperative	0 day (d)	Surgery	2 h	4 h	8 h	1 d	2d	3d	1 week (w)	Discharged	2w	4w	6w	3 months (m)	6 m
Enrollment																
Eligibility screening	X															
Informed consent	X															
Random allocation		X														
Interventions																
Betamethasone plus ropivacaine		X														
Ropivacaine		X														
Assessments																
Baseline data	X	X	X													
Intraoperative data			X													
Cumulative butorphanol consumption					X	X	X	X								
Patients with no butorphanol								X								
Total PCA button press count								X								
Time of first analgesia demand					X	X	X	X								
VAS_M_				X	X	X	X	X	X	X		X	X	X	X	X
VAS_R_				X	X	X	X	X	X	X		X	X	X	X	X
PSS				X	X	X	X	X	X	X		X	X	X	X	X
PONV				X	X	X	X	X								
RSS				X	X	X	X	X								
WHOQOL-BREF	X															X
ODI	X												X	X	X	X
POSAS																X
AEs																
Nausea				X	X	X	X									
Vomiting				X	X	X	X									
Gastritis				X	X	X	X									
GI bleeding				X	X	X	X									
Delayed wound healing				X	X	X	X									

### Sample size {14}

Ersayli et al. reported that total morphine consumption at 24 h was 13.2 ± 4.1 mg after lumbar discectomy for patients who received local wound infiltration of bupivacaine alone just before incision [14]. The analgesic effect of 1 mg morphine is the same as that of 3.75–7 mg butorphanol. This dose of morphine could be converted into an equianalgesic dose of butorphanol (with conversion factor 1 mg morphine = 3.75–7 mg butorphanol). Therefore, we have estimated that total butorphanol consumption will be about 120.0 ± 90.0 mg at 48 h after laminoplasty or laminectomy for the patients who received preemptive analgesia with local anesthetics.

Nakai et al. suggested that local wound infiltration by addition of betamethasone could reduce the dose of analgesics by about 45% in the 24 h after lumbar discectomy [18]. Therefore, we hypothesize that the cumulative butorphanol consumption during the 48 h after lumbar discectomy will be 120.0 ± 90.0 mg in the control group and 70.0 ± 50.0 mg in the treatment group. Based on a 90% power with a two-sided α of 0.05 and a dropout rate of 20%, we have calculated that at least 116 patients (58 per group) will be required.

### Recruitment {15}

The PRE-EASE trial team includes two research members from the department of neurosurgery who will be in charge of the patient recruitment process.

### Assignment of interventions: allocation

#### Sequence generation {16a}

Eligible participants will be randomly assigned by a computerized random-number list generator used for randomization (SPSS 25.0), after written consent is obtained.

#### Concealment mechanism {16b}

The study investigator will be responsible for preparing the respective drugs to be used for preemptive infiltration. Only the doctors in charge of the postoperative pain evaluation will be blinded, along with the outcome assessors and data analysts.

#### Implementation {16c}

An experienced sub-investigator, not involved in any other aspect of this study, will use SPSS 25.0 to generate a computerized random-number list, which will allocate participants to either one of the two groups. Participants who fulfill the inclusion criteria will be recruited by the neurosurgeons involved in the PRE-EASE trial. Eligible participants will be randomly assigned to their respective interventions according to the list generated by the computerized random-number list generator.

### Assignment of interventions: blinding

#### Who will be blinded {17a}

The doctors in charge of the postoperative pain evaluation will be blinded, along with the outcome assessors and data analysts.

#### Procedure for unblinding if needed {17b}

The design is open label with only the outcome assessors and data analysts being blinded, so unblinding will not occur.

### Data collection and management

#### Plans for assessment and collection of outcomes {18a}

The primary outcome of interest will be recorded by an electronic memory system, which will include both valid and invalid presses for butorphanol demand in the 48-h postoperative period. The online recording system of the PCA demand will only be accessible to the sub-investigators blinded to the study. Secondary outcomes will be postoperative patient-reported scores, collected by a group of blinded research members in charge of the postoperative pain evaluation. At completion of the 6 months’ follow-up data collection, we will perform a data quality audit. An investigator will sample every participant file and check for missing data.

#### Plans to promote participant retention and complete follow-up {18b}

Follow-up will be conducted on day 3, at weeks 1, 2, 4, and 6, and at months 3 and 6 by an experienced research member blinded to the study. All the participants will complete a 6 months’ follow-up. Follow-up data collection will either be done in person during the patient follow-up visits or by contact via telephone. Any participants who do not complete the entire 6 months’ follow-up process due to deviation from intervention, discontinuation for personal reasons, or failure of contact will not be replaced by other patients. Participants will be allowed to withdraw their consent or discontinue participation without any restriction at any time throughout the study, and further data associated with the trial will be collected.

#### Data management {19}

The primary outcome will be recorded in an online recording system accessible only to sub-investigators blinded to the study. Secondary outcomes will be postoperative patient-reported scores, collected by a group of blinded research members in charge of the postoperative pain evaluation. At completion of the 6 months’ follow-up data collection by a blinded research member, we will perform a data quality audit. All data collected will be stored in a secure location by the lead investigator, undisclosed to other research members.

#### Confidentiality {27}

All personal information about the participants will be collected and stored in a secure cabinet by the lead investigators, throughout the duration of the study, to guarantee confidentiality. Only the lead investigator will have access to the files corresponding to the personal data of the participants.

#### Plans for collection, laboratory evaluation, and storage of biological specimens for genetic or molecular analysis in this trial/future use {33}

This trial does not involve collecting, laboratory evaluation, or storage of biological specimens for genetic or molecular analysis.

### Statistical methods

#### Statistical methods for primary and secondary outcomes {20a}

Statistical analyses will be performed using the statistical package SPSS software 25.0. A Kolmogorov-Smirnov test will be used to assess normality of variables. Data for normal distribution will be presented as mean ± SEM (standard error of the mean). Variables for skewed distributions will be described as median and interquartile range (IQR). Categorical variables will be expressed as frequencies with percentages.

Comparisons between the groups will be carried out using an independent *t* test to compare normally distributed data, the Mann-Whitney *U* test for skewed data, and a χ^2^ test or Fisher’s exact test to compare categorical data such as safety analyses with the incidence of AEs. For numerical data collected at different time points throughout the course of 6 months (e.g., PCA cumulative consumption of butorphanol, PONV, RSS, PSS, and ODI), repeated measures analysis of variance will be performed between the two groups. The significance level will be set at *P* < 0.05.

#### Interim analyses {21b}

Although there are no anticipated problems that may be detrimental to the participants, serious life-threatening AEs leading to prolonged hospital stay or death will be reported to the Institutional Review Board (IRB), and our study will be terminated immediately.

#### Methods for additional analyses (e.g., subgroup analyses) {20b}

Prior to statistical analysis, a sub-investigator will review the data record forms to check for their legitimacy and identify the missing data. The subgroup analysis will be conducted to evaluate outcomes in patients based on their baseline clinical and demographic characteristics, such as gender, age, weight, and type of surgery (laminectomy or laminoplasty).

#### Methods in analysis to handle protocol non-adherence and any statistical methods to handle missing data {20c}

All researchers will be trained referring to the same training protocol. Protocol modifications will not be expected. Missing intraoperative data, if any, will be obtained from the electronic hospital files. Postoperative evaluation at specified time points is mandatory, and missing postoperative data are not to be anticipated. Analyses of all outcomes will be performed according to the intention-to-treat principle, and once enrolled, all participants will be analyzed, regardless of the findings.

#### Plans to give access to the full protocol, participant level-data, and statistical code {31c}

The data collected will be kept in a secure cabinet. Only the research members and the IRB of Beijing Tiantan Hospital will have access to the files. After the completion of the study, the results will be made public through publication in a scientific journal along with conferences related to neurosurgical anesthesia, as well as the ClinicalTrials.gov website. The data generated or analyzed during this study will be considered to be available from the corresponding author on reasonable request.

### Oversight and monitoring

#### Composition of the Coordinating Center and Trial Steering Committee {5d}

The Coordinating Center (CC) will comprise a Principal Investigator (PI), an expert on pain management, a neurosurgeon, a neurosurgical nurse, and a statistician. The CC will establish a communication network between the research members involved in the recruitment, preoperative evaluation, perioperative intervention, postoperative evaluation, and follow-up process. It will be responsible for the training of the research members regarding every aspect of the study protocol, along with the coordination of all standardized quality control aspects of the trial including the operations manual, forms, etc. The CC will also supervise data management, analysis, and publications of the study.

The Trial Steering Committee (TSC) will include the PI, an independent chair, two independent clinicians, an independent statistician, and representatives from the funding institution, who will oversee the work of various subcommittees. The subcommittee responsible for quality control will ensure standardized training for research members regarding the study protocol and their performance review. A subcommittee comprising the neurosurgical staff will oversee recruitment and clinical activities. Another subcommittee will oversee the review and approval of publications and presentations.

Participants will be recruited from the neurosurgical outpatient department at Beijing Tiantan Hospital, affiliated to Capital Medical University, Beijing, China. Based on the inclusion and exclusion criteria, patients will be screened for study participation by the subcommittee comprising the neurosurgical research members of the trial. Patients scheduled for surgery who fulfill the eligibility criteria and express an interest in participating in the study will be visited by a research assistant, 1 day before the surgery, to obtain written consent. A verbal explanation of the written consent will be provided by the research member, and any questions regarding the study will be answered. If the patients are willing to participate, written consent will be obtained, and patients will be taught how to indicate postoperative pain levels based on the VAS and how to use the PCA device when they feel pain.

The TSC will be responsible for overseeing the progress of the trial, for overall supervision, and for finding solutions to unforeseen problems that may arise in the course of the study.

The TSC will perform thorough assessment of the potential association between the study interventions and the AEs and will report to the IRB if necessary. The IRB of Beijing Tiantan Hospital will supervise the trial and will meet at least annually to oversee conduct and progress.

A Stakeholder and Public Involvement Group (SPIG) has not been appointed for this trial.

#### Composition of the Data Monitoring Committee, its role and reporting structure {21a}

An independent Data Monitoring Committee (DMC), responsible for independent review of participant safety and data endpoints, has been appointed. The independent members include two statisticians and a clinician. The DMC will report directly to the TSC at their meeting after every 25%, 50%, 75%, and 100% of patient inclusions.

#### Adverse event reporting and harms {22}

The IRB of Beijing Tiantan Hospital will conduct regular inspections of the trial progress. Any AEs will be recorded, and a thorough assessment of the potential association between the study interventions and the AE will be carried out. Serious life-threatening AEs leading to prolonged hospital stay or death will be reported to the IRB, and the PRE-EASE trial will be terminated immediately.

#### Frequency and plans for auditing trial conduct {23}

The IRB of Beijing Tiantan Hospital will make regular inspections of the trial conduct. The inspections will be independent of those of the investigators and the sponsor.

#### Plans for communicating important protocol amendments to relevant parties (e.g., trial participants, ethical committees) {25}

Although protocol amendments are not to be expected, any deviations from the protocol will be fully documented in a breach report form, reported to all regulatory bodies, and thoroughly recorded in a protocol deviation log. Protocol amendments will be first submitted to the sponsor within 7 days and then to the relevant parties by sending the updated protocol to the investigators. A copy of the revised protocol will be added to the Investigator Site File. The protocol will also be updated in the ClinicalTrials.gov registry website.

#### Dissemination plans {31a}

After the completion of the study, the results will be made public through publication in a scientific journal, at conferences related to neurosurgical anesthesia, and on the ClinicalTrials.gov website.

## Discussion

This trial will be a PROBE study. It will have better application in routine clinical practice, along with the clinical outcomes of a large simple research, which will permit a broader patient population and will include the advantage of randomization and an extensive evaluation of endpoints by blinded experts [19]. To our knowledge, there has been no attempt in the past to infiltrate laminoplasty or laminectomy with a preemptive local administration of betamethasone and ropivacaine. This study will add significant new knowledge to the effect and feasibility of preemptive wound infiltration of betamethasone.

Due to its large particles occluding the blood vessels supplying the spinal cord, betamethasone was previously reported to result in infarction of spinal cord after epidural analgesia [20]. However, we speculate that local peri-incisional infiltration of betamethasone is safe, as betamethasone has previously been used for intralesional [21], local infiltration [22], intramuscular [23], and intra-articular injections [16]. Local infiltration of steroid hormones will undeniably present the risk of delayed wound healing or local infection. However, we intend to use the lowest possible concentration of betamethasone for local infiltration based on the previous literature [15, 16, 22, 24], and thus this concentration should be considered safe. Wound healing and infection will be closely observed. The study will be immediately terminated in case of serious adverse reactions by the PRE-EASE Trial Management Group.

There are still some limitations regarding our study. Firstly, this is a single-center study; a multicenter study would be helpful in providing more significant data. In addition to incisional pain, acute pain after laminoplasty or laminectomy may be followed by long-term chronic pain that may not only originate from the incisional wound, but may also include neuropathic pain from spinal cord damage and nerve root injury. Another possible limitation of this study is that it will only involve infiltration of the surrounding tissue of the incision site. Therefore, we would like to suggest a further detailed study, regarding whether local betamethasone injection into the affected nerve roots before closure could be beneficial for postoperative pain after laminoplasty or laminectomy.

### Trial status

This research protocol version 2 (2019/12/22) is approved by the IRB of Beijing Tiantan Hospital, affiliated to Capital Medical University. Recruitment of patients for this PRE-EASE trial will begin in February of 2020, and is expected to complete by the end of 2021.

## Data Availability

After the completion and following the publication of the PRE-EASE trial, requests for data sharing will be considered by the PRE-EASE Trial Management Group.

## Abbreviations

AE: Adverse event
GI bleeding: Gastrointestinal bleeding
IRB: Institutional Review Board
ODI: Oswestry Disability Index
PCA: Patient-controlled analgesia
PONV: Postoperative nausea and vomiting
POSAS: Patient and Observer Scar Assessment Scale
PSS: Patient Satisfaction Score
RSS: Ramsay Sedation Scale
VAS_M_: Visual Analog Score during movement
VAS_R_: Visual Analog Score during rest
WHOQOL-BREF: World Health Organization Quality of Life-BREF
